# Cancer-associated fibroblast-secreted IGFBP7 promotes gastric cancer by enhancing tumor associated macrophage infiltration via FGF2/FGFR1/PI3K/AKT axis

**DOI:** 10.1038/s41420-023-01336-x

**Published:** 2023-01-21

**Authors:** Dandan Li, Lingyun Xia, Pan Huang, Zidi Wang, Qiwei Guo, Congcong Huang, Weidong Leng, Shanshan Qin

**Affiliations:** 1 Department of Stomatology, Taihe Hospital, Hubei University of Medicine, Shiyan, Hubei People’s Republic of China; 2 Hubei Key Laboratory of Embryonic Stem Cell Research, School of Basic Medical Sciences, Hubei University of Medicine, Shiyan, Hubei People’s Republic of China; 3 Laboratory of Tumor Biology, Academy of Bio-medicine Research, Hubei University of Medicine, Shiyan, Hubei People’s Republic of China

**Keywords:** Cancer microenvironment, Gastric cancer, Tumour immunology

## Abstract

We previously reported that IGFBP7 plays a role in maintaining mRNA stability of oncogenic lncRNA UBE2CP3 by RNA-RNA interaction in gastric cancer (GC). Clinical cohort studies had implied an oncogenic role of IGFBP7 in GC. However, the molecular mechanism of IGFBP7 in GC progression remains unknown. In this study, clinical analysis based on two independent cohorts showed that IGFBP7 was positively associated with poor prognosis and macrophage infiltration in GC. Loss-of-function studies confirmed the oncogenic properties of IGFBP7 in regulating GC cell proliferation and invasion. Mechanismly, IGFBP7 was highly expressed in cancer-associated fibroblasts (CAF) and mesenchymal cells, and was induced by epithelial-to-mesenchymal transition (EMT) signaling, since its expression was increased by TGF-beta treatment and reduced by overexpression of OVOL2 in GC. RNA sequencing, qRT-PCR, ELISA assay showed that IGFBP7 positively regulated FGF2 expression and secretion in GC. Transcriptome analysis revealed that FGFR1 was downregulated in M1 polarization but upregulated in M2 polarization. Exogenous recombinant IGFBP7 treatment in macrophages and GC cells further identified that IGFBP7 promotes tumor associated macrophage (TAM) polarization via FGF2/FGFR1/PI3K/AKT axis. Our finding here represented the first evidence that IGFBP7 promotes GC by enhancing TAM/M2 macrophage polarization through FGF2/FGFR1/PI3K/AKT axis.

## Introduction

Gastric cancer (GC) is the third most common heterogeneous tumor with the highest mortality rate in the world [1]. Due to the inability of early diagnosis of GC, most patients diagnosed at advanced stage, leading to poor prognosis. Metastasis is the leading cause of cancer-related death worldwide [2, 3]. The most common route of GC metastasis is lymph node metastasis, followed by peritoneal dissemination metastasis and liver metastasis [4, 5]. Approximately one-third of GC patients are diagnosed at an advanced stage with metastasis, and 4–14% have metastatic disease to the liver [6, 7]. It is urgently necessary to unravel the molecular mechanisms underlying metastasis.

The insulin-like growth factor binding protein (IGFBP) family contains seven members, including IGFBP1–7 [8]. These IGFBPs modulate the actions of the insulin-like growth factors in endocrine, paracrine, and autocrine settings [9]. Unlike the other six IGFBPs which bind IGFs with high affinity, IGFBP7 binds IGF-I and IGF-II with relatively low affinity [10]. Recent advances have shown that IGFBP7 appears to exhibit IGF-independent biological effects in tumor progression. For example, IGFBP7 plays tumor-suppressive roles in melanoma, thyroid carcinogenesis, and breast cancer by induction of cell senescence and apoptosis [11–13].

The biological function of IGFBP7 in GC was rarely studied. Recently, two independent clinical cohort studies have implied an oncogenic role of IGFBP7 in GC, since IGFBP7 overexpression predicted poor prognosis in GC [14, 15]. Likewise, our previous study also implied an oncogenic role of IGFBP7 in GC by positively regulating oncogenic lncRNA UBE2CP3 expression [16]. However, these studies do not provide direct evidence of the oncogenic properties of IGFBP7. In addition, the molecular mechanism by which IGFBP7 promotes GC progression remains unclear.

Increasing evidence have shown that tumor associated macrophage (TAM) infiltration play critical roles in tumorigenesis and metastasis [17–19]. Recently, fibroblast growth factor 2 (FGF2) has been reported to promote macrophage polarization towards an M2/TAM phenotype [20]. Knockout of FGF2 in mice altered macrophage polarization towards an inflammatory (M1) phenotype [20]. Takase et al. reported that FGF2 promotes TAM infiltration and tumor progression in esophageal squamous cell carcinomas through FGF2/FGFR1 axis [21]. Similarly, Qin and colleagues found that serine protease PRSS23 promotes GC progression by positively regulating FGF2 secretion to enhance TAM infiltration [22]. In this study, we identified a novel role of IGFBP7 in regulating TAM infiltration by enhancing FGF2 secretion and activating FGF2/FGFR1/PI3K/AKT signaling.

## Results

### IGFBP7 overexpression was clinically associated with poor prognosis in GC

**Fig. 1:**
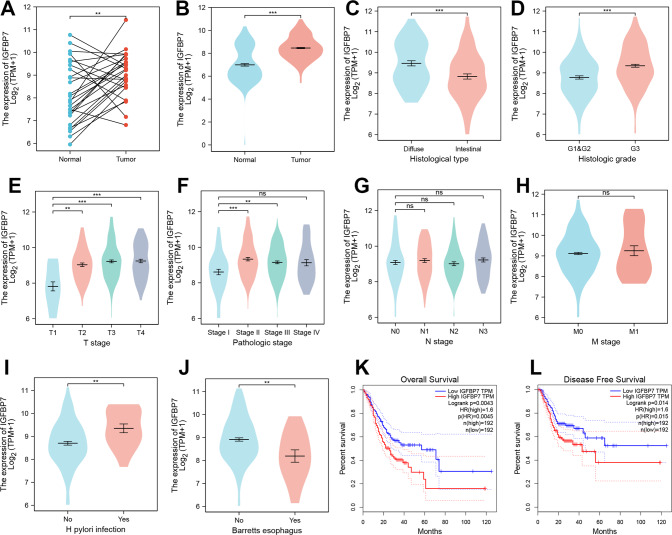
The prognostic significance of IGFBP7 in GC was analyzed in TCGA cohort. **A** IGFBP7 expression in 27 paired GC samples in TCGA_STAD datasets. **B** IGFBP7 was overexpressed in GC. **C** The expression difference of IGFBP7 in intestinal and diffuse GC. **D** IGFBP7 expression was positively correlated with poor differentiation of GC. **E**–**H** The expression level of IGFBP7 in GC tissues with different TNM stages and pathologic stages. **I** The expression difference of IGFBP7 in GC patients with/without H. pylori infection. **J** GC patients with Barrett’s esophagus possessed relatively low expression of IGFBP7. **K**, **L** The overall-survival and disease-free survival analysis of IGFBP7 in GC. **P < 0.01.

Two independent large cohorts of GC were included in this study, including the TCGA_STAD cohort (*n* = 373) and the ACRG cohort (GSE62254, *n* = 300). The TCGA_STAD cohort contains 27 paired of GC samples. After analyzing the expression data in 27 pairs of GC samples, it was found that IGFBP7 was significantly upregulated in GC (Fig. 1A, *p* < 0.01). Likewise, IGFBP7 was overexpressed in GC by comparing the normalized expression data in the GTEx_stomach cohort (normal) and TCGA_STAD cohort (Fig. 1B, *p* < 0.001).

To uncover the clinical significances of IGFBP7 overexpression in GC, we first analyzed the correlation between gene expression and the clinical features of GC in TCGA cohort. The results showed that diffuse GC patients or patients with relatively poorer degree of differentiation (G3 stage) had higher expression of IGFBP7 (Fig. 1C, D). Although the high expression of IGFBP7 was significantly correlated with T stage and pathological stage of GC, it was not significantly associated with N stage and M stage (Fig. 1E–H). Interestingly, GC patients with positive H. pylori infection (Hp) or with negative Barrett’s esophagus tended to possess relatively high expression of IGFBP7 (Fig. 1I, J). Consistently, GC patients with higher expression of IGFBP7 had a shorter OVS and DFS time (Fig. 1K, L).

**Fig. 2:**
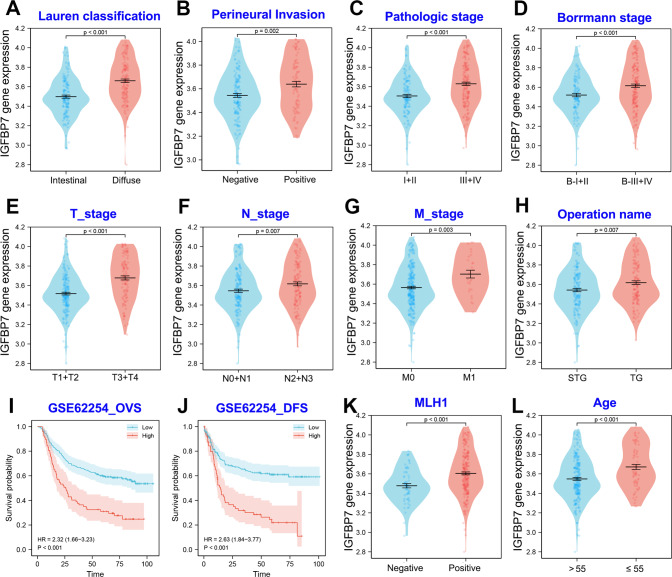
The clinical value of IGFBP7 was analyzed in ACRG cohort. **A** The expression difference of IGFBP7 in intestinal and diffuse GC. **B** The expression difference of IGFBP7 between GC patients with/without perineural invasion. **C**, **D** The expression difference of IGFBP7 in GC patients with different pathologic stages or Borrmann stages. **E**–**G** The expression level of IGFBP7 in GC tissues with different TNM stages. **H** The expression difference of IGFBP7 between GC patients with pylorus-sparing radical gastrectomy (STG) operation or total gastrectomy (TG) operation. **I**, **J** The overall-survival and disease-free survival analysis of IGFBP7 in GC. **K** The expression difference of IGFBP7 between GC patients with/without MLH1 expression. **L** The expression difference of IGFBP7 between GC patients over 55 years old and patients under 55 years old. **P < 0.01.

Similarly, the clinical value of IGFBP7 overexpression was further verified in the ACRG cohort (GSE62254). IGFBP7 overexpression showed a significant correlation with the histopathological type, perineural invasion, malignant progression (including TNM stages, pathologic stage and Borrmann stage), surgical approach and poor prognosis of GC (Fig. 2A–J). Notably, GC patients with positive MLH1 immunohistochemical staining or younger age possessed higher IGFBP7 expression (Fig. 2K, L). Taken together, IGFBP7 can serve as a biomarker the diagnosis and prognosis of GC.

### IGFBP7 expression was regulated by EMT signaling in GC

**Fig. 3:**
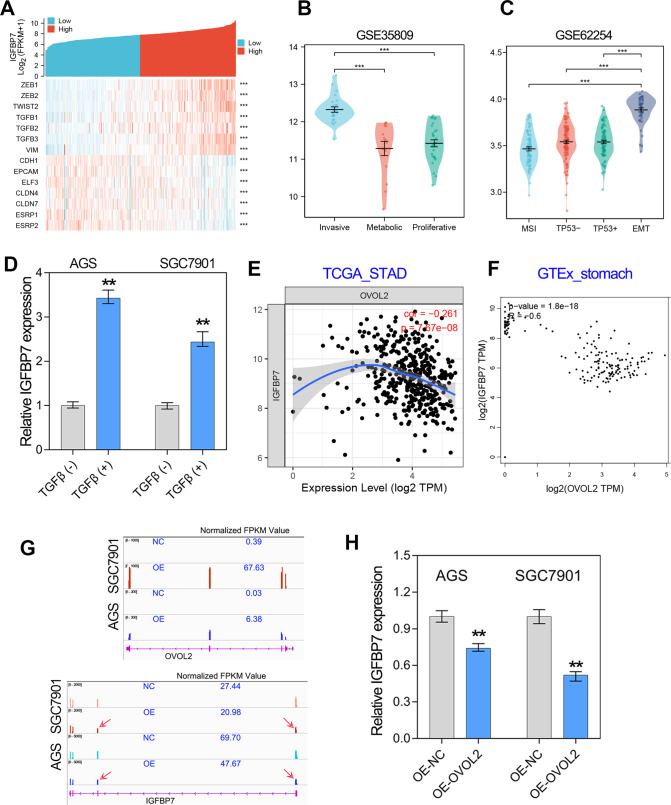
IGFBP7 expression was positively regulated by EMT signaling in GC. **A** Gene expression correlation analysis confirmed that IGFBP7 was related to EMT signaling in GC. **B**, **C** Expression level of IGFBP7 in the different subtypes of GC in GSE35809 and GSE62254 cohorts. **D** The expression of IGFBP7 was determined by qRT-PCR after TGF-beta treatment. **E**, **F** The expression correlation between IGFBP7 and OVOL2 in GC tissues and normal stomach tissues. **G** RNA-seq analysis was conducted in GC cells overexpressing OVOL2. IGFBP7 expression was decreased in GC cells overexpression OVOL2. **H** Quantitative RT-PCR assay showed that the expression of IGFBP7 was significantly reduced by OVOL2 in GC. ****P < 0.0001; ***P < 0.001; **P < 0.01; *P < 0.05.

To figure out why IGFBP7 was overexpressed in GC, we conducted expression correlation analysis between IGFBP7 and other genes using RNA-seq data of TCGA. The result showed that IGFBP7 was positively correlated with the expression of mesenchymal biomarker genes, but negatively correlated with the expression of epithelial genes in GC (Fig. 3A, *p* < 0.001). Based on gene expression profiling features, Lei et al. have classified GC patients in GSE35809 cohort (*N* = 70) into 3 subtypes, including proliferative, metabolic and mesenchymal [23]. Cristescu and colleagues reported that GC could be further divided into four subtypes, including MSS/TP5−, MSS/TP53+, MSI and MSS/EMT subtypes [24]. After analysis of IGFBP7 expression in different subtypes of 2 cohorts, we noted that IGFBP7 was relatively high expressed in mesenchymal subtype of GSE35809 cohort and EMT subtype of GSE62254 cohort (Fig. 3B, C, *p* < 0.001). That means IGFBP7 might be highly associated with EMT signaling in GC.

To further determine whether IGFBP7 is responsive to the EMT signaling, we examined the expression level of IGFBP7 in GC cells with TGF-beta treatment or OVOL2 overexpression. TGF-beta was known to be a critical cellular EMT-inducing signal molecule [25]. Our previous study has successfully constructed GC cells with a mesenchymal phenotype by treatment with exogenous TGF-beta [26]. After detecting the gene expression by qRT-PCR, we found that IGFBP7 expression was greatly induced by TGF-beta in GC (Fig. 3D). In addition, we previously constructed GC cells with stable overexpression of OVOL2 [26]. Given IGFBP7 expression was significantly negatively correlated with OVOL2 expression in both GC tissues and normal stomach tissues (Fig. 3E, F), we further investigated the IGFBP7 expression level in GC cell lines by qRT-PCR assay and RNA-seq analysis. The results showed that overexpression of OVOL2, a well-known MET inducer, significantly decreased the mRNA level of IGFBP7 in GC cell lines (Fig. 3G, H). These data together indicated that IGFBP7 is induced by EMT signaling in GC.

### Knockdown of IGFBP7 inhibits GC cell proliferation and invasion in vitro and in vivo

**Fig. 4:**
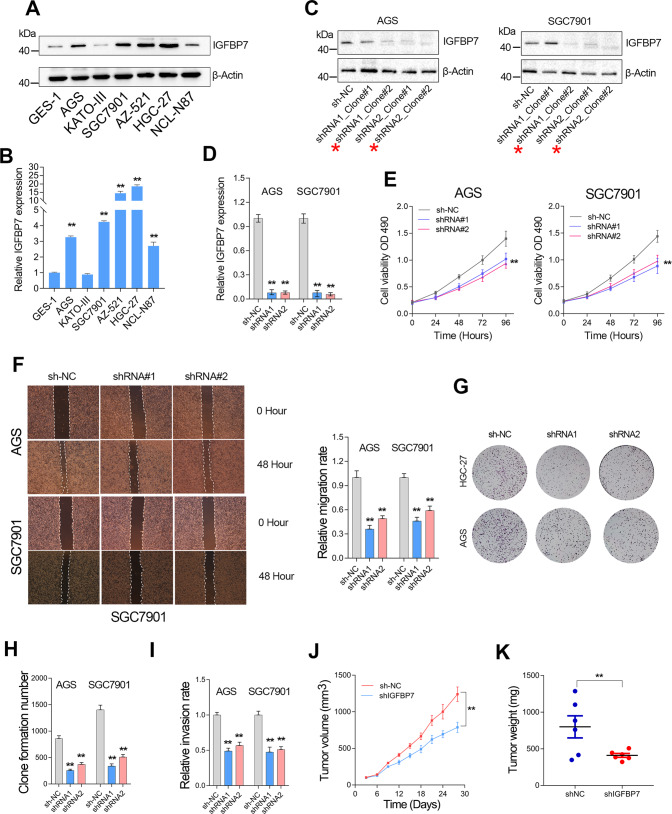
IGFBP7 knockdown inhibited GC cell proliferation, migration and invasion. **A**, **B** IGFBP7 expression level in different GC cell lines was examined by western blotting and qRT-PCR assays. **C**, **D** The knockdown efficiency of IGFBP7 in GC cell lines was determined by western blotting and qRT-PCR assays. **E** The growth rate of GC cells was determined by MTT assay after knockdown of IGFBP7 in GC cell lines. **F** The migration of GC cells was examined by wound healing assay after knockdown of IGFBP7 in GC cell lines. **G**, **H** The proliferation of GC cells was examined by clone formation assay after knockdown of IGFBP7 in GC cell lines. **I** The transwell invasion of GC cells was examined after knockdown of IGFBP7 in GC cell lines. **J** Tumor volumes for the indicated Day 3 to 30 after injecting shNC and shIGFBP7 cells into nude mice. Data represent mean tumor volumes ± SEM. **K** After 30 days of subcutaneous tumor bearing, nude mice were euthanized. The subcutaneous transplanted tumor in nude mice was taken out and weighed. **P < 0.01.

The above results had implied IGFBP7 functioned as an oncogene in GC. Thus, loss-of-function studies were further performed to validate the effects of IGFBP7 on the biological behavior of GC cells. First, we detected the protein and mRNA expression of IGFBP7 in different GC cell lines. The results showed that IGFBP7 was highly expressed in most GC cell lines (Fig. 4A, B). Herein, we selected AGS and SGC7901 GC cell lines for lentiviral knockdown of IGFBP7. Two different short hairpin RNAs (shRNA) targeting IGFBP7 were used in loss-of-function studies to avoid off-target effects. To validate the knockdown efficiency of IGFBP7, we selected two single clones from each of the lentiviral-transfected GC cell lines for western blotting validation (Fig. 4C). The optimal clone for each shRNA in two GC cell lines were used in further experiments (Fig. 4D). Cell proliferation assay showed that IGFBP7 knockdown suppressed the cell growth of GC cell lines (Fig. 4E). The wounding healing assay showed that knockdown of IGFBP7 suppressed the cell migration of GC cell lines (Fig. 4F). Clone formation assay showed that depletion of IGFBP7 hindered the proliferation of GC cells (Fig. 4G, H). Transwell assay indicated that IGFBP7 knockdown significantly repressed the metastasis of GC cells (Fig. 4I). Likewise, the in vivo assays also found that IGFBP7 plays a tumor-promoting and pro-metastatic role in GC (Fig. 4J, K).

### IGFBP7 depletion repressed FGF2 expression and secretion

**Fig. 5:**
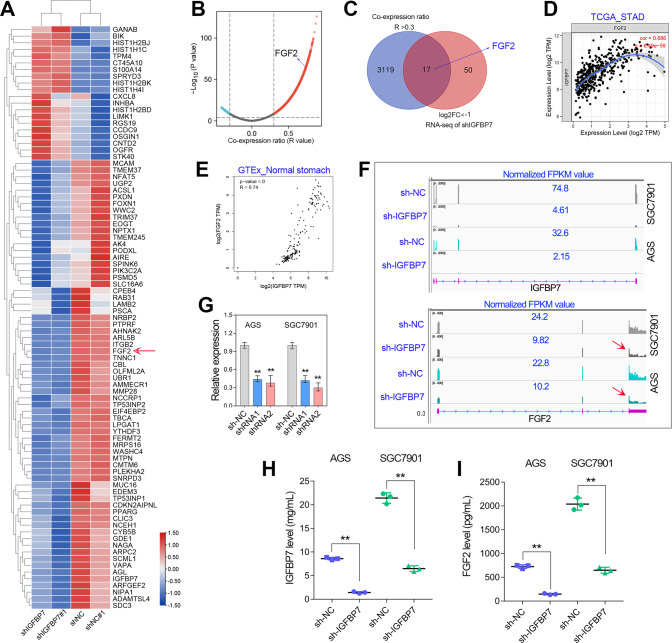
IGFBP7 depletion suppressed FGF2 expression and secretion in GC. **A** The differentially expressed genes after IGFBP7 depletion were shown in the heatmap. Among them, FGF2 was downregulated by knockdown of IGFBP7 in GC. **B** The expression correlation analysis between protein-coding genes and IGFBP7 was conducted in GC. **C** A total of 17 genes that co-expressed with IGFBP7 were greatly downregulated by IGFBP7 depletion in GC cell lines. **D**, **E** IGFBP7 was highly co-expressed with FGF2 in both GC tissues and normal stomach tissues. **F**, **G** RNA-seq and qRT-PCR analysis showed that IGFBP7 depletion decreased the expression of FGF2. **H**, **I** ELISA assay verified that IGFBP7 knockdown decreased secreted level of IGFBP7 and FGF2. **P < 0.01.

Our above finding suggests that IGFBP7 plays an essential role in promoting GC progression, however the underlying molecular mechanism remains unclear. Therefore, transcriptome sequencing studies were further performed in GC cell lines (Fig. 5A). After RNA-seq analysis, the genes with the most significant fold change in expression after IGFBP7 depletion were shown in the heat map (Fig. 5A). On the other hand, we also screened the potential genes highly co-expressed with IGFBP7 (*R* > 0.3) by expression correlation analysis. After taking insertion, a total of 17 genes were identified to be positively regulated by IGFBP7 in GC, including human fibroblast growth factor 2 (Fig. 5B, C). Consistently, FGF2 and IGFBP7 were highly co-expressed in both GC and normal stomach tissues (Fig. 5D, E). RNA-seq analysis and qRT-PCR assay showed that IGFBP7 depletion decreased FGF2 mRNA level in GC cell lines (Fig. 5F, G). Enzyme-linked immunosorbent assay (ELISA) showed that knockdown of IGFBP7 decreased the secreted level of IGFBP7 and FGF2 (Fig. 5H, I). Given FGF2 is clinically associated with poor prognosis in GC (Fig. S1a–l), we speculate that IGFBP7 may promote GC progression by enhancing FGF2 expression and secretion.

### CAFs secreted IGFBP7 promotes TAM infiltration by regulating FGF2 secretion

**Fig. 6:**
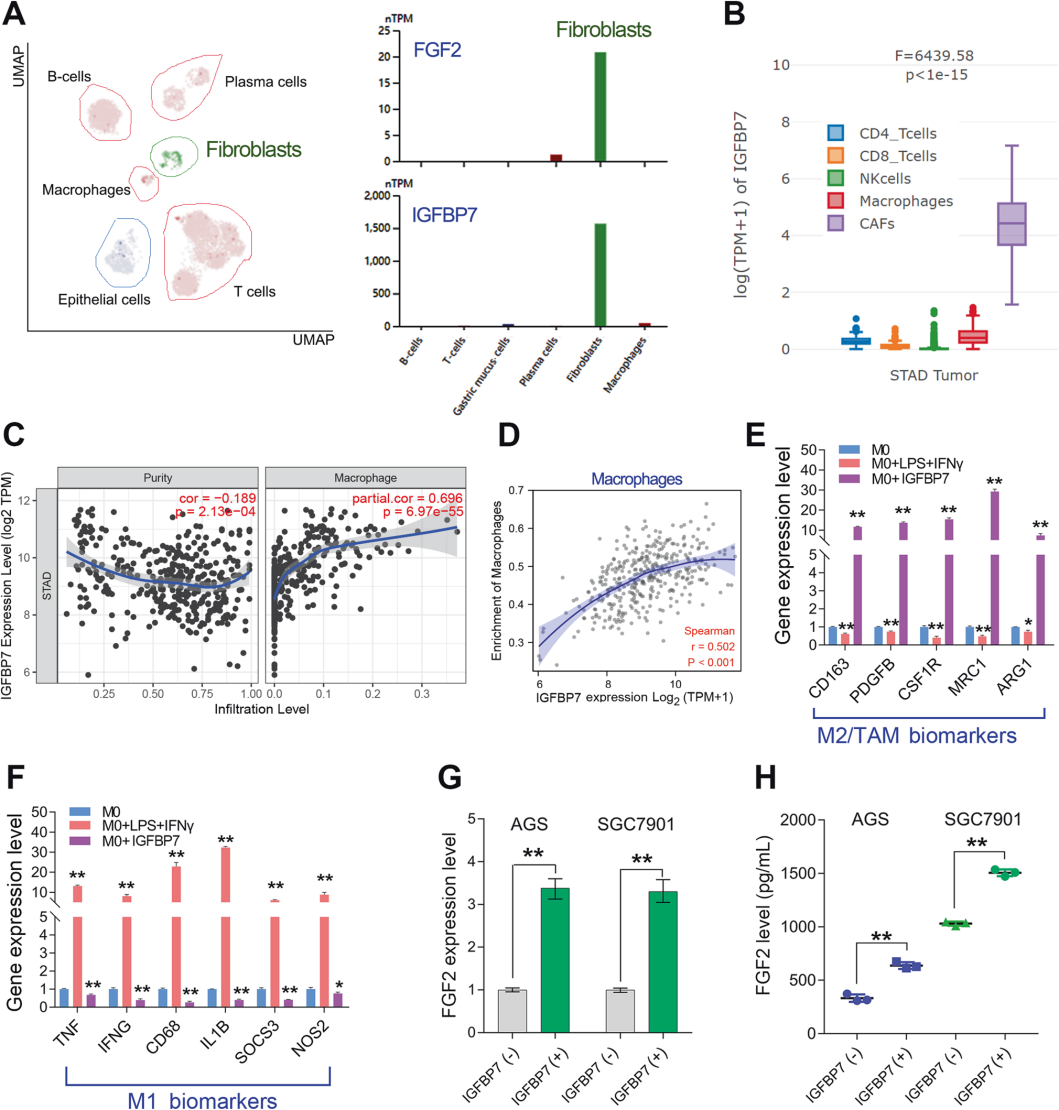
IGFBP7 promotes TAM infiltration and FGF2 expression in GC. **A** Single-cell analysis confirmed that IGFBP7 and FGF2 was specifically expressed in fibroblasts. **B** Immune estimation using EPIC method showed that IGFBP7 was highly expressed in CAFs of GC. **C**, **D** Immune infiltration analysis using TIMER or CIBERSOTR showed that IGFBP7 expression was highly associated with macrophage infiltration in GC. **E**, **F** Exogenous IGFBP7 treatment in macrophages downregulated the expression of M1 macrophage biomarkers, but upregulated expression of M2/TAM macrophage biomarkers. **G**, **H** Exogenous IGFBP7 treatment in GC cells upregulated FGF2 expression and secretion. **p < 0.01.

To verify the cell-type-specificity of IGFBP7 in GC, single-cell analysis based on HPA dataset was used. The single-cell analysis in HPA datasets contains 9 cell subsets, including fibroblast, T cells, B cells, plasma cells, macrophages and gastric epithelial cells. Both IGFBP7 and FGF2 were specifically expressed in the fibroblast (Fig. 6A). Immune estimation using EPIC algorithm further confirmed IGFBP7 was highly expressed in CAFs (Fig. 6B). Previous studies have reported that FGF2 plays critical roles in macrophage infiltration and polarization [22, 27–31]. Thus, we analyzed the correlation between IGFBP7 expression and immune infiltration using two methods, including CIBERSORT and TIMER. Both of the 2 algorithms confirmed that IGFBP7 was positively correlated with macrophage infiltration level in GC (Fig. 6C, D).

To further explore the effect of IGFBP7 on macrophage polarization, we treated GC cell lines with exogenous recombinant IGFBP7 protein, as previously reported [11]. Firstly, the M0 macrophages were derived from THP-1 cells by treatment with PMA. Then, M1 macrophages with pro-inflammatory phenotype were induced by treatment with LPS and IFNG as described previously [32]. After determination the expression level of M1 or M2/TAM macrophage biomarker genes, we noted that IGFBP7 treatment partially suppressed the expression of M1 macrophage biomarkers but enhanced the expression level of M2/TAM macrophage biomarker genes (Fig. 6E, F). These results indicate that IGFBP7 promotes macrophage polarization towards a M2/TAM phenotype. Previous study has reported a critical role of FGF2 on TAM polarization and infiltration [20]. Correspondingly, we further detected the expression level and secreted level of FGF2 after IGFBP7 treatment. The results showed that IGFBP7 treatment enhanced FGF2 expression and secretion in GC cell lines (Fig. 6G, H). Taken together, IGFBP7 promotes TAM infiltration by promoting FGF2 expression and secretion in GC.

### IGFBP7 promotes tumor associated macrophage infiltration through FGF2/FGFR1/PI3K/AKT axis

**Fig. 7:**
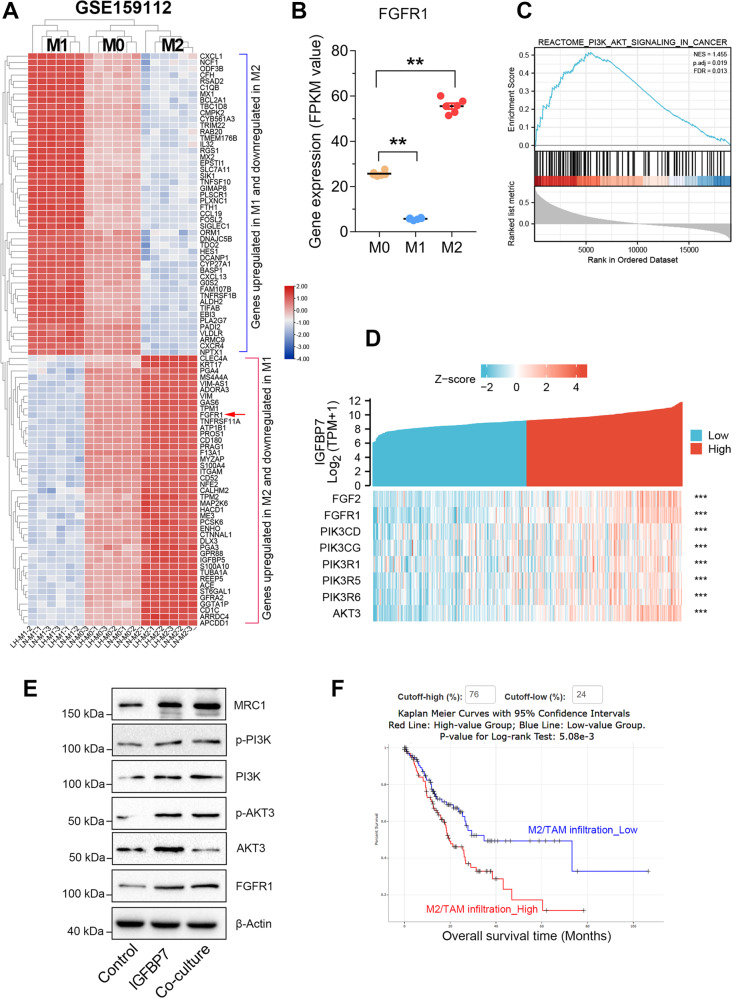
IGFBP7 promotes TAM infiltration by activating FGFR1/PI3K/AKT signaling. **A** Differentially expressed genes of macrophages polarized towards M1 phenotype or towards M2 phenotype. **B** FGFR1 was significantly downregulated in M1 macrophages but upregulated in M2 macrophages. **C**, **D** The GSEA and expression correlation analysis showed that IGFBP7 was highly associated with PI3K/AKT signaling in GC. **E** Exogenous IGFBP7 promotes TAM/M2 macrophages polarization via FGFR1/PI3K/AKT signaling. **F** High level of M2/TAM macrophage infiltration predicted poor prognosis in GC. **p < 0.01.

Although Rizzo and colleagues have demonstrated a critical role of FGF2 in macrophage polarization in knockout mice [20], the underlying molecular mechanism remains largely unclear. To uncover how FGF2 promotes tumor associated macrophage infiltration, we compared the transcriptome of M0, M1, and M2 macrophages using GSE159112 datasets [33]. The genes involved in regulating macrophage polarization are shown in the heat map (Fig. 7A). The genes simultaneously downregulated in M1 (log2FC < −1) and upregulated in M2 (log2FC > 1) are thought to be involved in macrophage polarization towards a M2/TAM-like phenotype. Among them, there is a well-known receptor of fibroblast growth factor, (FGFR1) that is greatly overexpressed during M2 polarization but under-expressed during M1 polarization (Fig. 7B).

Increasing evidence have reported FGFR1 can directly activate PI3K/AKT signaling by binding to its ligands, such as FGF2 [34–36]. Therefore, we speculated IGFBP7 may promote M2/TAM-like macrophage polarization by FGF2/FGFR1/PI3K/AKT axis. The GSEA analysis confirmed the closely association between PI3K/AKT signaling and IGFBP7 expression in GC (Fig. 7C). Besides, gene expression correlation analysis showed that IGFBP7 was highly co-expressed with the genes involved into FGF2/FGFR1/PI3K/AKT signaling (Fig. 7D). More importantly, western blotting assay showed that either IGFBP7 treatment or co-culture with GC cells activated the PI3K/AKT signaling and enhanced macrophage polarization towards to a M2/TAM phenotype (Fig. 7E). These results together indicated that IGFBP7 enhanced M2/TAM-like macrophage polarization via FGF2/FGFR1/PI3K/AKT axis.

**Fig. 8:**
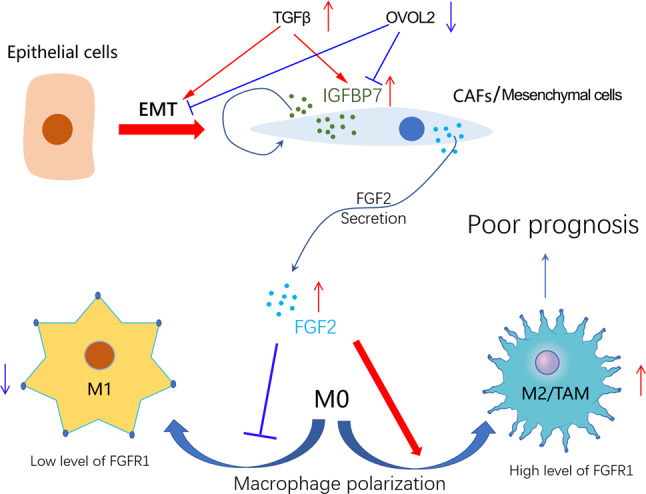
Working model of IGFBP7 in promoting macrophage infiltration and GC progression. Abnormal EMT signaling led to overexpression of IGFBP7 in CAFs. Meanwhile, IGFBP7 overexpression increased the levels of IGFBP7 and FGF2 in tumor microenvironment. Eventually, FGF2 promotes TAM polarization and infiltration through FGFR1/PI3K/AKT axis, thereby resulted in poor prognosis of GC.

In addition, survival analysis showed that high level of M2/TAM infiltration predicted poor prognosis in GC (Fig. 7F). Thus, we drew a working model that IGFBP7 may promote GC progression by enhancing TAM macrophage polarization via FGF2/FGFR1/PI3K/AKT axis (Fig. 8). Briefly, dysregulated EMT signaling mediated by TGF-beta or other EMT-related factors resulted in increased level of CAFs and IGFBP7 overexpression. Then, IGFBP7 overexpression further enhanced the expression and secretion of FGF2 in CAFs. The increased level of FGF2 in tumor microenvironment promotes TAM polarization and infiltration through FGFR1/PI3K/AKT axis, thereby resulting in poor prognosis of GC patients.

## Discussion

The role of IGFBP7 in tumor progression is complicated, since IGFBP7 exhibits opposite roles in different cancers. It has been IGFBP7 was reported to play tumor suppressive role in breast cancer, thyroid carcinogenesis and melanoma by induction of cell apoptosis and cell senescence [11–13]. While, clinical cohort studies have implied an oncogenic role of IGFBP7 in oesophagus cancer and stomach cancer [14, 15, 37–40]. However, the molecular mechanism of IGFBP7 in promoting the GC progression remains unknown to date.

As a member of the IGFBP family with relatively low affinity to IGF-1 and IGF-II, IGFBP7 appears to play roles in tumor progression in an IGF-independent manner. Several independent studies have uncovered a closely association between IGFBP7 and PI3K/AKT signaling. Zhang et al. reported that IGFBP7 inhibits thyroid carcinoma by regulating AKT activity and cell cycle progression [41]. Similarly, Chen et al. reported that IGFBP7 acted as an upstream molecule of AKT3/mTOR signaling [42]. Diao et al. found that YTHDF2 promotes GBM progression by regulating IGFBP7/PI3K/AKT signaling [43]. Niu et al. revealed that PI3K/AKT signaling was activated by overexpression of IGFBP7 in AML [44].

In this study, clinical analysis based on two independent cohorts showed that IGFBP7 overexpression was clinically associated with poor prognosis in GC. Loss-of-function studies confirmed that IGFBP7 depletion inhibits GC proliferation and invasion in vivo and in vitro. More importantly, our finding first uncovered a novel role of IGFBP7 in TAM infiltration. Immune infiltration analysis showed that IGFBP7 was highly correlated with macrophage infiltration. Exogenous recombinant IGFBP7 treatment on M0 macrophages decreased the expression level of the biomarkers in M1 macrophages with a pro-inflammatory phenotype and increased the expression level of biomarkers in M2/TAM-like macrophages with a pro-tumor phenotype. These results indicated that IGFBP7 promotes macrophage polarization towards a M2/TAM phenotype in GC.

Furthermore, our work highlights that IGFBP7 promotes M2/TAM macrophage infiltration via FGF2/FGFR1/PI3K/AKT axis. Firstly, RNA-seq analysis and ELISA studies verified a promoting effect of IGFBP7 on the expression and secretion of FGF2. Secondly, transcriptome analysis verified that FGFR1 played critical roles in M2/TAM polarization, since its expression was downregulated during M1 polarization but upregulated during M2 polarization. Coincidentally, FGF2 was reported to play essential roles in M2/TAM macrophage polarization [29–31]. Thirdly, exogenous IGFBP7 treatment in macrophages activated FGFR1/PI3K/AKT signaling. Increasing studies have reported the critical role of PI3K/AKT signaling in M2/TAM macrophage infiltration [45–47]. Moreover, FGFR1 is one of the important membrane receptors upstream of the PI3K/AKT axis [34]. Once FGFR1 recognized FGF2, it can directly initiate the PI3K/AKT axis [36, 48, 49]. Consistently, macrophage co-cultured with GC cells or exposure to IGFBP7 greatly increased the expression of MRC1, a well-known of M2/TAM biomarker gene, and activated FGFR1/PI3K/AKT signaling. Taken together, IGFBP7 promotes TAM/M2 macrophage polarization through FGF2/FGFR1/PI3K/AKT axis.

TAM/M2 infiltration level can be served as a prognostic biomarker of GC [50–52]. Higher level of TAM/M2 infiltration predicted poor prognosis in GC (Fig. 7F). Similarly, TAM/M2 infiltration contributed to the progression in gastric cancer with peritoneal dissemination [53]. Our finding here represented the first evidence that IGFBP7 promotes GC by enhancing TAM/M2 macrophage polarization through FGF2/FGFR1/PI3K/AKT axis.

## Materials and methods

### Clinical cohort study

The gene profile data and corresponding clinical information of GC patients were obtained from the Cancer Genome Atlas (TCGA) database. The expression level of per gene was calculated from log2 of TPM value. Clinical information of patients in the GSE62254 cohort was obtained from Cristescu and colleagues [24]. The gene expression array data of GSE62254 cohort was downloaded from the Gene Expression Omnibus (GEO) database on the NCBI web server.

### Mouse xenograft model

Four-week-old female BALB/c nude mice used in this study were maintained in the animal facility of the Laboratory Animal Center of Hubei University of Medicine. All animals were treated in accordance with guidelines of the Committee on Animals of the Hubei University of Medicine. The mice were randomly divided into two groups (shNC and shIGFBP7). The corresponding GC cell lines were cultured as usual and were injected into the subcutaneous tissue of nude mice. After a month, all the mice were euthanized. The xenograft tumors were collected for weighing and volume measurement. The study protocol was approved by the Experimental Animal Research Ethics Committee of Hubei University of Medicine (2021-028).

### Immune infiltration analysis

The GEPIA 2021 web tool (http://gepia2021.cancer-pku.cn/index.html) provides multiple immune estimation algorithms, including CIBERSORT, EPIC, and quanTIseq [54]. These algorithms were used to evaluate the correlation between genes and different types of immune cells in pan-cancer. The CIBERSORT method provides the immune estimation of 24 immune cell types. The EPIC method was conducted to analyze the expression level of IGFBP7 in different Immune cells, including CAFs, CD4/8 T-cells, B cells, NK cells macrophages and endothelial cells. Survival analysis of different macrophage infiltration was performed using quanTIseq method. The TIMER method was used to estimate the correlation between different genes and the level of immune infiltration of macrophages in TCGA cohort. The gene expression level in different immune cell types between stomach cancer and normal stomach tissues and the survival analysis of M2/TAM infiltration were analyzed using GEPIA 2021 web tool.

### Cell culture and establishment of lentiviral cell lines

All GC cell lines used in this study were cultured in DMEM medium containing 10% fetal bovine serum (FBS) at 37 °C in 5% CO_2_. The lentiviruses for knockdown of IGFBP7 were purchased from GeneChem (Shanghai, China). Monoclonal cell lines were obtained by serial density dilution of contemporary transfected cells. After verifying the knockdown efficiency of IGFBP7 by WB and qRT-PCR, an optimal monoclonal cell line for each shRNA was selected for subsequent experiments.

### Recombinant IGFBP7 or TGF-beta treatment

For recombinant IGFBP7 protein treatment, the GC cells were grown as usual and plated into the six-well plates. When the cells adhered to the wall, the recombinant IGFBP7 protein (2 μg/mL, cat. no. ab50195; Abcam, Shanghai, China) was added to the medium. For recombinant TGFβ protein treatment, the GC cells were treated with 10 ng/ml recombinant TGF-β1 protein (cat. no. HZ-1011; Proteintech, Wuhan, China) [55]. At the indicated time points, the cells were harvested for mRNA and protein analysis as well as for other assays.

### Macrophage induction

The THP-1 cell line was treated with PMA (Sigma, 100 ng/mL) for 24 h to induce M0 macrophages. Next, the THP-1 derived M0 macrophages were plated into a six-well transwell plate. Meanwhile, the digested HGC-27 cell suspension was added to the 0.4 μm porous membrane of upper chamber. The THP-1-derived macrophages were co-cultured with HGC-27 cells for 48 h. Macrophages derived from THP-1 cells were treated with recombinant IGFBP7 protein for 48 h. At the indicated time points, macrophages were collected for RNA extraction and other experiments.

### RNA sequencing

Total RNA of IGFBP7 stable knockdown cell lines was extracted and sent to Lifegenes company (Shanghai, China) to perform RNA sequencing. The RNA-seq data (Table S1) used in this study has been uploaded in the GEO dataset (GSE163813 and GSE207373).

### Quantitative RT-PCR assay

The quantitative RT-PCR assay was performed as previously described [56]. The specific primers for qRT-PCR assay were designed and synthesized by Wcgene Biotech (Shanghai, China). The relative gene expression level was determined by 2^–∆∆Ct^ method.

### Western blotting assay

The western blotting assay was performed as previously described [55, 57]. The antibodies of IGFBP7 (A2982), FGF2 (A11488), AKT (60203-2-Ig), p-AKT (28731-1-AP), MRC1 (A11192), and PI3K (20584-1-AP) were purchased from Abclonal company (Wuhan, China) and Proteintech company (Wuhan, China). The Phospho-PI3K antibody was purchased from Cell Signaling Technology (13857S, MA, USA).

### Statistical analysis

The *P* values for IGFBP7 expression analysis in GC cohorts were estimated using Mann–Whitney nonparametric test. The *P* values of survival analysis were estimated using the log-rank test. Correlation analysis was used to test the correlation between the two groups of data. The *P* values for qRT-PCR assay were estimated using ANOVA method. *P* < 0.05 considered statistically significant.

## Supplementary information

Supplementary Figure legends

Supplemental Figure S1

Table S1

Original Data File

Authorship change form

## Data Availability

All data are available upon request.
