# Supplementary material for: Cancer-associated fibroblast-secreted IGFBP7 promotes gastric cancer by enhancing tumor associated macrophage infiltration via FGF2/FGFR1/PI3K/AKT axis
Source: Cell Death Discov. 2023 Jan 21;9:17. doi: 10.1038/s41420-023-01336-x (PMC9867714; doi:10.1038/s41420-023-01336-x)
Supplement: Supplementary file 1 — Supplementary Figure legends [file 41420_2023_1336_MOESM1_ESM.docx]

**Figure S1.** The clinical value of FGF2 was analyzed in ACRG cohort. **(A)** The expression difference of IGFBP7 in intestinal and diffuse GC. **(B)** The expression difference of FGF2 between GC patients with/without MLH1 expression. **(C)** The expression difference of FGF2 between GC patients over 55 years old and patients under 55 years old. **(D)** The expression difference of FGF2 between GC patients with/without perineural invasion. **(E-I)** The expression difference of FGF2 in GC patients with different TNM stages, pathologic stages or Borrmann stages. **(K)** The expression difference of FGF2 between GC patients with pylorus-sparing radical gastrectomy (STG) operation or total gastrectomy (TG) operation. **(I, J)** The overall-survival and disease-free survival analysis of FGF2 in GC.
