# Supplementary figures and images for: Cancer-associated fibroblast-secreted IGFBP7 promotes gastric cancer by enhancing tumor associated macrophage infiltration via FGF2/FGFR1/PI3K/AKT axis
Source: Cell Death Discov. 2023 Jan 21;9:17. doi: 10.1038/s41420-023-01336-x (PMC9867714; doi:10.1038/s41420-023-01336-x)

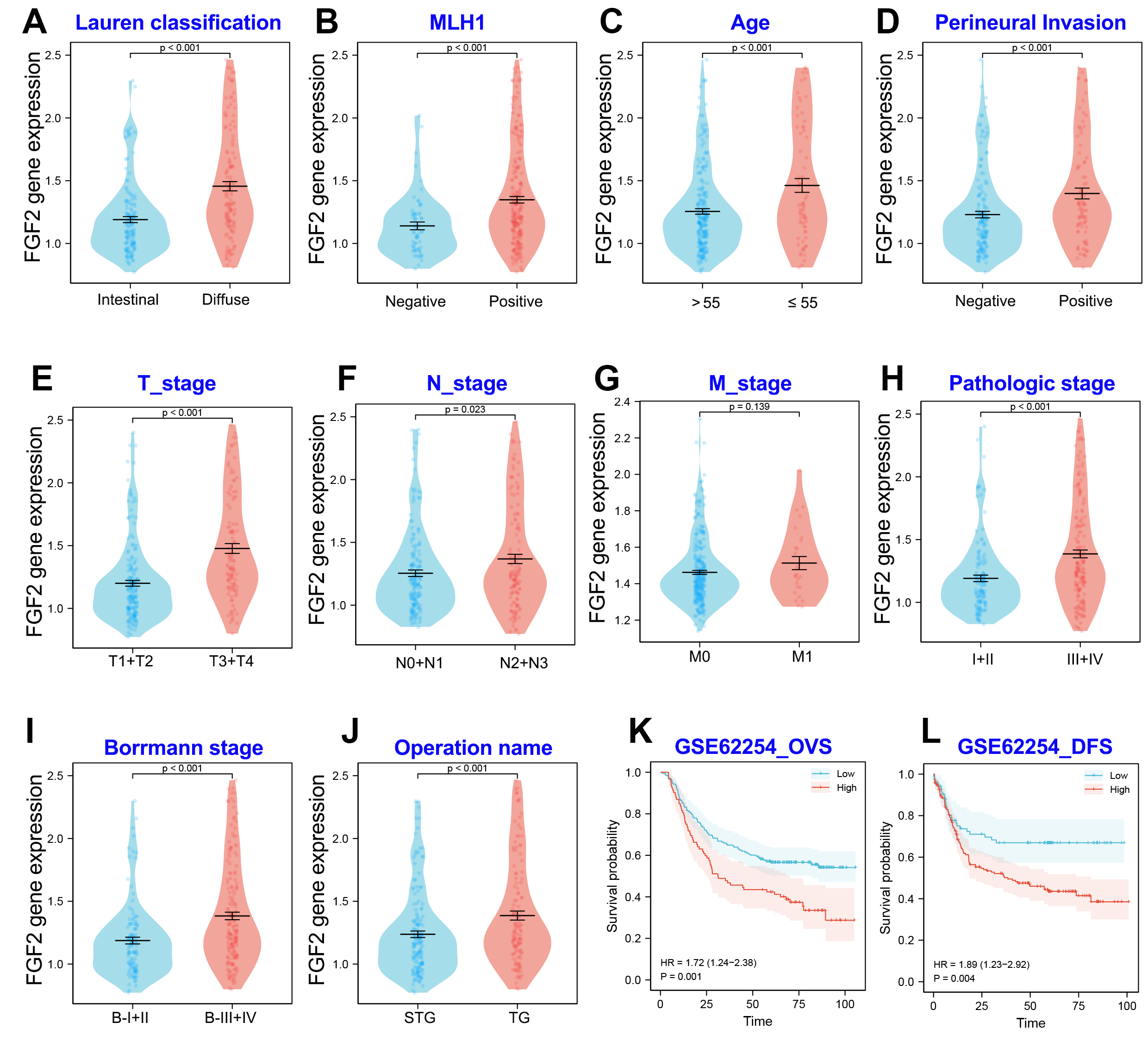

Supplement: Supplementary file 2 — Supplemental Figure S1 [file 41420_2023_1336_MOESM2_ESM.tif]

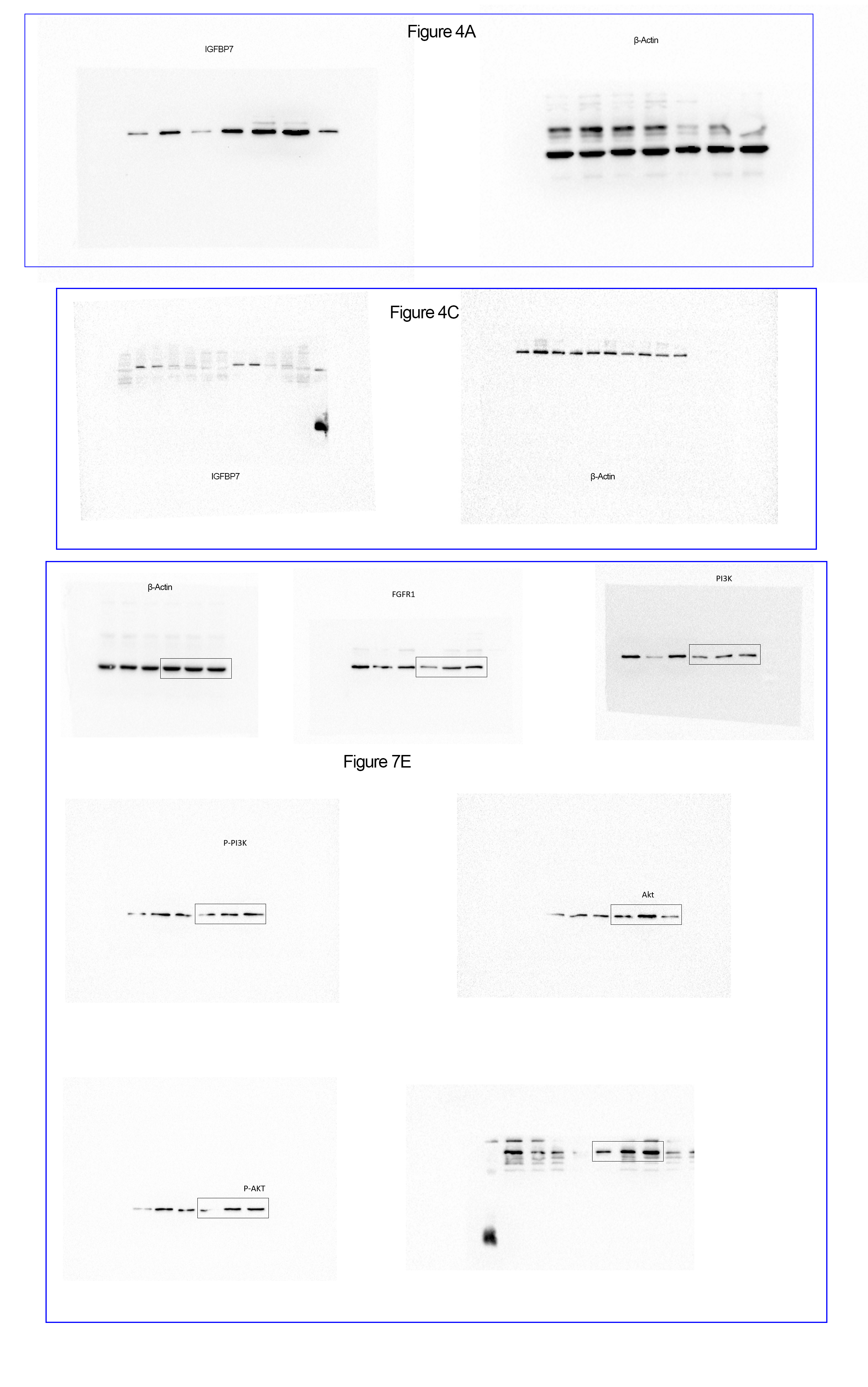

Supplement: Supplementary file 4 — Original Data File [file 41420_2023_1336_MOESM4_ESM.tif]
