# Supplementary material for: Cancer-associated fibroblast-secreted IGFBP7 promotes gastric cancer by enhancing tumor associated macrophage infiltration via FGF2/FGFR1/PI3K/AKT axis
Source: Cell Death Discov. 2023 Jan 21;9:17. doi: 10.1038/s41420-023-01336-x (PMC9867714; doi:10.1038/s41420-023-01336-x)
Supplement: Supplementary file 5 — Authorship change form [file 41420_2023_1336_MOESM5_ESM.pdf]

Changes to the author list

Manuscript No. CDDISCOVERY-22-5557

Article Entitled: Cancer-associated fibroblast-secreted IGFBP7 promotes gastric cancer by enhancing tumor associated macrophage infiltration via FGF2/FGFR1/PI3K/AKT axis

Author information:

Dandan Li [1,2,3]#, Lingyun Xia [1]#, Pan Huang [2,3]#, Zidi Wang [3], Qiwei Guo [3], Congcong Huang [2,3], Weidong Leng [1]* and Shanshan Qin [1,2,3]*

[1] Department of Stomatology, Taihe Hospital, Hubei University of Medicine, Shiyan 442000, Hubei, P.R. China

[2] Hubei Key Laboratory of Embryonic Stem Cell Research, School of Basic Medical Sciences, Hubei University of Medicine, Shiyan 442000, Hubei, P.R. China

[3] Laboratory of Tumor biology, Academy of Bio-Medicine Research, Hubei University of Medicine, Shiyan 442000, Hubei, P.R. China

# These authors contributed equally to this work.

* **Corresponding author:**

Shanshan Qin, Email: qinss77@163.com; Weidong Leng, Email: lwd35@163.com

Dear authors:

Because of the contributions by XLY and LWD in the revision stage, we need to change the current manuscript’s author order and addresses, as listed above.

Among the authors in the list, QSS designed experiments, offered direction and help on the whole project. LDD, XLY, WZD, HP, HCC and GQW conducted the experiments, analyzed the results. QSS and LDD performed bioinformatics analysis and drafted the manuscript. QSS and LWD reviewed the manuscript and made significant revisions on the drafts. All authors read and approved the final manuscript..

After consultations, all the authors agreed with the addition of authors in this paper, and all the authors agreed with the rearrangement of the names.

If everyone have no comments on the change of authorship of this manuscript, please email to me with "I agree to these changes".

Thank you very much for your attention, looking forward to your reply.

Best Wishes

Correspondence to Shanshan Qin, Ph. D.

Hubei Key Laboratory of Embryonic Stem Cell Research

Hubei University of Medicine

Renmin Road 30, Maojian District

Shiyan, Hubei 442000, P.R. China

Changes to the author list（CDDISCOVERY-22-5557）

发件人：qinss77<qinss77@163.com>

收件人：lidandan cup<lidandan_cup@163.com> hbmuwzd<hbmuwzd@163.com> panhuang<panhuang@hbmu.edu.cn> huangcong163<huangcong163@126.com> hbmugqw<hbmugqw@163.com> xialingyun200810<xialingyun200810@163.com> lwd35<lwd35@163.com> ↑隐藏信息 保存所有收件人

时　间：2023年01月12日 20:23 (星期四)

发送状态：发送成功 查看详情

翻译成中文

您的邮箱安全待提升！仅需1分钟，安全性提升30%，一键升级>>

Changes to the author list

Manuscript No. CDDISCOVERY-22-5557

Article Entitled: Cancer-associated fibroblast-secreted IGFBP7 promotes gastric cancer by enhancing tumor associated macrophage infiltration via FGF2/FGFR1/PI3K/AKT axis

Author information:

Dandan Li [1,2,3]#, Lingyun Xia [1]#, Pan Huang [2,3]#, Zidi Wang [3], Qiwei Guo [3], Congcong Huang [2,3], Weidong Leng [1]* and Shanshan Qin [1,2,3]*

Re: Changes to the author list（CDDISCOVERY-22-5557）

发件人：李丹丹<lidandan_cup@163.com>

收件人：qinss77<qinss77@163.com>

时　间：2023年01月12日 20:35 (星期四)

I agree to these changes.

lidandan_cup@163.com

邮箱：lidandan_cup@163.com

---- Replied Message ----

From	qinss77<qinss77@163.com>
Date	01/12/2023 20:23
To	lidandan_cup@163.com<lidandan_cup@163.com>、hbmuwzd@163.com<hbmuwzd@163.com>、panhuang@hbmu.edu.cn<panhuang@hbmu.edu.cn>、huangcong163@126.com<huangcong163@126.com>、hbmugqw@163.com<hbmugqw@163.com>、xialingyun200810@163.com<xialingyun200810@163.com>、lwd35@163.com<lwd35@163.com>

Re:Changes to the author list（CDDISCOVERY-22-5557）

发件人：夏凌云<xialingyun200810@163.com>

收件人：qinss77<qinss77@163.com>

时　间：2023年01月12日 22:24 (星期四)

您的邮箱安全待提升！仅需1分钟，安全性提升30%，一键升级>>

I agree to these changes.

Re:Changes_to_the_author_list（CDDISCOVERY-22-5557）

发件人：黄盼<panhuang@hbmu.edu.cn>

收件人：qinss77<qinss77@163.com> +

时　间：2023年01月12日 20:30 (星期四)

您的邮箱安全待提升！仅需1分钟，安全性提升30%，一键升级>>

I agree to these changes

---------- Origin message ----------

>From："qinss77" <qinss77@163.com>

>To：lidandan_cup@163.com, hbmuwzd@163.com, panhuang@hbmu.edu.cn, huangcong163@126.com, hbmugqw@163.com, xialingyun200810@163.com, lwd35@163

>Subject：Changes_to_the_author_list（CDDISCOVERY-22-5557）

>Date：2023-01-12 20:23:04

Changes to the author list

Re: Changes to the author list（CDDISCOVERY-22-5557）

发件人：hbmuwzd@163.com<hbmuwzd@163.com>

收件人：qinss77<qinss77@163.com> +

时　间：2023年01月12日 22:50 (星期四)

翻译成中文

您的邮箱安全待提升！仅需1分钟，安全性提升30%，一键升级>>

I agree to these changes.

---- Replied Message ----

From	qinss77<qinss77@163.com>
Date	01/12/2023 20:23
To	lidandan_cup@163.com<lidandan_cup@163.com>、hbmuwzd@163.com<hbmuwzd@163.com>、panhuang@hbmu.edu.cn<panhuang@hbmu.edu.cn>、huangcong163@126.com<huangcong163@126.com>、hbmugqw@163.com<hbmugqw@163.com>、xialingyun200810@163.com<xialingyun200810@163.com>、lwd35@163.com<lwd35@163.com>
Subject	Changes to the author list（CDDISCOVERY-22-5557）

Re:Changes to the author list（CDDISCOVERY-22-5557）

发件人：hbmugqw<hbmugqw@163.com>

收件人：qinss77<qinss77@163.com> +

抄送人：lidandan cup<lidandan_cup@163.com> hbmuwzd<hbmuwzd@163.com> panhuang<panhuang@hbmu.edu.cn> huangcong163<huangcong163@126.com> xialingyun200810<xialingyun200810@163.com>

抄送人

时　间：2023年01月12日 20:37 (星期四)

您的邮箱安全待提升！仅需1分钟，安全性提升30%，一键升级>>

I agree to these changes.

Re:Changes to the author list（CDDISCOVERY-22-5557）

发件人：冷卫东<lwd35@163.com>

收件人：qinss77<qinss77@163.com> +

时　间：2023年01月12日 22:10 (星期四)

您的邮箱安全待提升！仅需1分钟，安全性提升30%，一键升级>>

I agree to these changes.

Re: Changes to the author list（CDDISCOVERY-22-5557）

发件人：聪聪<huangcong163@126.com>

收件人：qinss77<qinss77@163.com> +

时 间：2023年01月12日 20:27 (星期四)

翻译成中文

您的邮箱安全待提升！仅需1分钟，安全性提升30%，一键升级>>

I agree to these changes.

---- Replied Message ----

From	qinss77<qinss77@163.com>
Date	01/12/2023 20:23
To	lidandan_cup<lidandan_cup@163.com>,<br>hbmuwzd<hbmuwzd@163.com>,<br>panhuang<panhuang@hbmu.edu.cn>,<br>huangcong163<huangcong163@126.com>,<br>hbmugqw<hbmugqw@163.com>,<br>xialingyun200810<xialingyun200810@163.com>,<br>lwd35<lwd35@163.com>
Subject	Changes to the author list（CDDISCOVERY-22-5557）
